# Basic Principles of Skin Biopsy Optimization in Dermatopathology

**DOI:** 10.3390/dermatopathology13030042

**Published:** 2026-09-08

**Authors:** Mar Llamas-Velasco, Eduardo Rozas-Muñoz, Angel Fernandez-Flores, Maria-Teresa Fernandez-Figueras

**Affiliations:** 1 Department of Dermatology, Hospital de la Princesa, 28006 Madrid, Spain; mar.llamasvelasco@gmail.com; 2 Department of Dermatology, Hospital San Pablo, Coquimbo 1781881, Chile; docrozas@yahoo.com; 3 Department of Cellular Pathology, Hospital Universitario El Bierzo, 24404 Ponferrada, Spain; dermatopathonline@gmail.com; 4 Department of Anatomic Pathology, Hospital Universitari General de Catalunya, Grupo Quironsalud, Sant Cugat del Vallés, 08195 Barcelona, Spain; 5 Department of Medicine, Faculty of Medicine and Health Science, Universitat Internacional de Catalunya, Sant Cugat del Vallés, 08195 Barcelona, Spain

**Keywords:** skin biopsy, dermatopathology, histopathology, biopsy technique, quality, diagnostic accuracy, clinicopathological correlation, specimen handling, pre-analytical, pitfalls

## Abstract

Skin biopsy is one of the most valuable diagnostic procedures in dermatology, particularly when clinical findings alone are insufficient to establish a diagnosis. However, obtaining an accurate histopathological diagnosis depends on multiple steps, and errors at any stage of the biopsy pathway may compromise the final result. This review synthesizes current evidence and available guidelines on best practices for skin biopsy, integrating the practical experience of four internationally recognized dermatopathologists to address areas where evidence is limited or poorly standardized. The review covers biopsy planning, selection of the optimal biopsy site and technique, specimen handling and fixation, grossing and laboratory processing, prevention of technical artifacts, the use of ancillary diagnostic techniques, and clinicopathological correlation, with particular attention to challenging anatomical sites and complex diseases. Diagnostic accuracy depends on obtaining a representative specimen, maintaining high technical standards throughout tissue processing, providing adequate clinical information, and ensuring close communication between the clinician and the dermatopathologist. In selected cases, multidisciplinary review is required to reach a definitive diagnosis. Adherence to these principles can optimize diagnostic yield, reduce avoidable errors, and ultimately improve patient care.

## Simple Summary

Skin biopsy is one of the most valuable diagnostic procedures in dermatology, particularly when clinical findings alone are insufficient to establish a diagnosis. However, obtaining an accurate histopathological diagnosis depends on multiple steps, and errors at any stage may compromise the final result. This review combines current evidence with the practical experience of four internationally recognized dermatopathologists to provide a practical guide to optimizing skin biopsy procedures. It covers the selection of the most appropriate biopsy site and technique, proper specimen handling and fixation, practical aspects of grossing and laboratory processing, and the importance of good communication between the clinician and the dermatopathologist. We summarize best practices throughout the entire diagnostic pathway, from planning the biopsy to clinicopathological correlation, highlighting common pitfalls and practical recommendations to maximize diagnostic yield. Adherence to these principles can reduce avoidable errors, improve diagnostic accuracy, and ultimately enhance patient care.

## 1. Introduction

Dermatopathology requires precision and excellence throughout the entire diagnostic process, and the aim of this review is to provide a practical clinicopathological guide for clinicians, dermatopathologists, and histopathology laboratory managers. This introductory review presents general principles and practical recommendations to maximize the diagnostic information obtained from tissue specimens, improve diagnostic accuracy, and strengthen communication between clinicians and dermatopathologists. Specific topics are further expanded upon in the accompanying articles of this Special Issue, addressing inflammatory dermatoses, neoplasia, alopecia, pediatric dermatopathology, genital and nail pathology, immunofluorescence, as well as a comprehensive review of in vivo and ex vivo diagnostic techniques and other related topics.

The dermatopathological diagnostic process begins with the clinician, who is responsible for selecting the most representative lesion, obtaining and handling the specimen appropriately, and submitting adequate clinical information to the laboratory. At the histopathology laboratory, gross examination may include inking or derm-dotting [1] to identify areas of particular interest. Following tissue dehydration and paraffin embedding, laboratory technicians prepare paraffin blocks. Specialized histotechnologists are subsequently responsible for producing thin, even sections suitable for microscopic examination and digitalization. This task can be particularly challenging in specimens containing hyperkeratotic areas or marked variations in tissue hardness. Later, dermatopathologists should recognize incidental findings and artifacts introduced before the specimen reaches the laboratory or during tissue processing. Finally, while some cases allow a diagnosis to be established with a high degree of diagnostic certainty, others show non-specific histopathological findings that are only consistent with a particular entity or that help exclude suspected clinical diagnoses.

## 2. Materials and Methods

This is narrative review synthesizes core dermatopathology literature, standard practice guidelines, and practical diagnostic experience regarding skin biopsy handling. As a non-systematic review, formal search protocols, systematic selection criteria, and risk-of-bias tools were not utilized; instead, established clinical principles and guideline recommendations have been internalized and integrated throughout the text.

## 3. Results

### 3.1. General Considerations: Why Specimen Quality Matters

The quality of the specimen is one of the most important factors determining the value of the procedure. A well-performed biopsy not only confirms the clinician’s suspicion but may also clarify the diagnosis when the clinical impression is uncertain or incorrect. In simple terms the quality of the sample defines the limits of what the dermatopathologist can interpret. Providing an adequate specimen along with appropriate clinical information can increase diagnostic certainty from 53% to over 78% [2]. Conversely, inadequate sampling remains a leading cause of non-specific or purely descriptive pathology reports, which may account for up to 20% of skin biopsies in some series [3].

Several critical factors determine biopsy quality, including site selection, technical execution, and the choice of technique (punch, shave, or excision), among others [4]. For example, in autoimmune bullous diseases, incorrect site selection may result in false-negative direct immunofluorescence (DIF) findings, while in suspected melanoma, partial biopsies can compromise accurate staging [4].

A high-quality biopsy demands a thorough understanding of the procedure. Biopsies performed by trained physicians have been associated with higher diagnostic accuracy compared with those obtained by untrained physicians, reinforcing the importance of expertise in lesion selection and procedural technique [5].

### 3.2. Indications and Functions of Skin Biopsy

The main indications and functions of skin biopsy [6] are:

- Diagnosis: confirmation or exclusion of inflammatory and neoplastic dermatoses.
- Prognostic or staging: determination of critical parameters, such as the Breslow index in melanoma.
- Management and follow-up: defining surgical margins, monitoring drug efficacy, or detecting adverse effects.
- Medical safety: providing legal support and improving the doctor-patient relationship through objective data.

Skin biopsy is usually not recommended when the diagnosis of an inflammatory dermatoses is clinically obvious and the procedure would provide no new information, or when the risk of the procedure outweighs the benefit or can worsen the patient prognosis (e.g., severe vascular compromise in the lower leg where biopsy might induce a non-healing ulcer or in cases of suspected deep soft tissue sarcoma, where an inappropriate biopsy technique can induce tumor dissemination).

### 3.3. The Biopsy Pathway: From Biopsy Acquisition to Diagnosis

The biopsy pathway encompasses the sequence of actions or steps required to obtain a biopsy report. These can be divided into three major stages: pre-analytical, analytical, and post-analytical [7]. The pre-analytical stage includes clinical decision-making, lesion site and surgical technique selection, immediate fixation in the appropriate transport medium, and shipment to the histopathology laboratory. The analytical stage encompasses all laboratory processing, eventual digitization, and microscopic evaluation. The post-analytical stage includes to the preparation of the diagnostic report, which should reach the requesting clinician as soon as possible. The pathology report should include all relevant information and should be comprehensible to clinicians, sometimes with notes complementing or explaining the diagnosis. Clinicopathological discussion may occur at this stage, either at the initiative of the dermatopathologist or the clinician, or in clinicopathological conferences. These discussions are often extremely valuable and may lead to refinement or modification of the initial histopathological diagnosis. However, clinicopathological correlation is not confined to the post-analytical phase and may also contribute directly to diagnostic reasoning during the analytical phase. The increasing involvement of patients in their own healthcare, together with the common difficulty of understanding medical terms or details beyond the comprehension of the general population, could in the future be addressed by providing additional reports in plain language specifically designed for them [8].

Each of these stages involves numerous steps including communication exchanges, technical events, and cognitive decisions, all of which may affect the biopsy quality. It has been estimated that from the initial decision to biopsy to the final report, approximately 20 handoffs may occur [9]. Even assuming a 99% reliability at each individual step, the cumulative reliability of the entire process decreases to approximately 82%, highlighting that every phase carries a risk of error. Understanding this pathway as an integrated system is essential to minimizing avoidable diagnostic failure.

### 3.4. Clinical Information

#### 3.4.1. Relevant Clinical Information: What the Dermatopathologist Needs to Know

Submission of a skin biopsy without an adequate patient’s history may lead to important mistakes. Histopathological interpretation requires clinicopathological correlation. The famous quote from Claude Bernard, “The experimenter who does not know what he is looking for will never understand what he finds,” can be applied in this context.

Although, there is still no consensus on the exact critical information necessary for a proper histopathological evaluation, the dermatopathologist requires specific clinical context to correctly interpret the limited morphological patterns of the skin [10]. To ensure diagnostic accuracy, clinicians should avoid the use of vague terminology such as “skin rash, “papules,” “lesion,” or “recent changes” without any further specification of number, size or timing, as well as non-standard abbreviations [11]. The addition of clinical images alongside the biopsy is encouraged to improve this correlation, as clinical pictures of the patient’s skin lesions can often provide information that is difficult to convey in text alone [12].

#### 3.4.2. Mandatory Clinical Data and Lesion-Specific Information

At a minimum, the pathology requisition form should include the patient’s demographic data, such as age and sex and, where relevant, travel history, geographic origin, and skin color as certain diseases are strongly associated with specific demographic groups or geographic areas. Mentioning relevant symptoms such as pain and pruritus and significant personal and family history is also recommended.

Specifying the precise anatomic localization is critical, since normal histology varies according to body site. For example, a diagnosis of lichen simplex chronicus may be acceptable on the shin but requires exclusion of other entities on the scalp or genital region. Similarly, when the histology resembles palmar or plantar skin but hair follicles are present, the diagnosis of lichen simplex chronicus should be considered. Likewise, in the evaluation of melanocytic lesions, anatomic location is very important, as certain architectural and cytologic features that might be concerning elsewhere can be characteristic of specific areas such as acral, genital, or flexural skin (“special site nevi”), where site-specific criteria are required to avoid overdiagnosis of melanoma [13].

The clinician should also describe the primary morphology of the lesion (macule, papule, nodule, vesicle, or bulla) and the number of lesions, since using the plural “papules” implies that at least two are present, whereas the patient may in fact have hundreds. The size of the lesions and secondary changes such as ulceration or scale are also important to mention. Color and surface alterations, as well as the distribution pattern (localized, generalized, photo-exposed, acral), may significantly aid the differential diagnosis. Even when the clinical presentation is minimal or the signs are subtle, these findings should still be reported.

This is particularly important in so-called “invisible dermatoses,” which may account for up to 10% of biopsies and can present with subtle histopathological findings. Conditions such as tinea incognita or urticaria may be easily overlooked if the dermatopathologist is not prompted to actively search for fungal elements or subtle dermal edema [14].

#### 3.4.3. Temporal and Treatment-Related Data

Information regarding onset (congenital, childhood, recent), duration, lesion evolution (stable, progressive, episodic) and prior treatments is also important. For instance, entities such as lymphomatoid papulosis and mycosis fungoides may show similar histological features, and therefore the final diagnosis depends mainly on the clinical information.

Whenever possible, the timing of the biopsy in relation to previous treatments should be specified. For instance, topical corticosteroids may reduce the inflammatory infiltrate and mask diagnostic features, particularly in inflammatory disorders such as bullous pemphigoid or drug eruptions. Although this has not been systematically studied, topical corticosteroids are often recommended to be discontinued 2–3 weeks before biopsy, to avoid diagnostic errors [15]. Likewise, systemic treatments can modify inflammatory patterns and even alter immunohistochemical findings. A typical example is a patient with tumoral mycosis fungoides previously treated with brentuximab who may relapse with transformation but loses CD30 expression, as a consequence of therapy. A similar scenario may occur in B-cell lymphomas after rituximab treatment. Similarly, metastatic melanoma treated with anti-BRAF therapies may exhibit treatment-related cytologic changes.

#### 3.4.4. Clinical Differential Diagnosis

Providing the suspected clinical diagnosis along with relevant differential diagnoses, preferably in order of probability, significantly improves accuracy, especially in inflammatory disorders. This ensures that the pathology report provides a precise diagnosis, rather than simply a descriptive one. For example, in Fox-Fordyce disease, the most typical histopathological change is the presence of small aggregates of foamy macrophages around follicles [16]. This subtle finding is difficult to identify since it occurs in the context of non-specific follicular inflammation and sometimes requires the examination of multiple step sections to find the representative areas. Without proper clinical orientation, these changes may easily be missed, and the histopathological diagnosis could be simply folliculitis.

Additionally, specifying the principal diagnostic considerations may also guide the dermatopathologist to perform ancillary histochemical and immunohistochemical techniques. For instance, in a “pseudolymphomatous” dermal lymphoid hyperplasia with expansion of the marginal zone, knowing that the patient has risk factors for sexually transmitted diseases makes it advisable to perform an immunohistochemical study to detect *Treponema pallidum*, allowing the diagnosis of this rare form of secondary syphilis [16].

In oncological patients, knowing the type and current status of previous neoplasms can help identify metastases. In general, it is necessary to mention whenever a neoplasm is suspected, since this can prompt additional levels or closer histopathological evaluation if it is not identified in the initial sections. This is particularly true in entities such as lentigo maligna or acral melanocytic lesions, where small specimens or amelanotic lesions may cause false-negative results. Similarly, in alopecia, indicating the suspected subtype (scarring versus non-scarring) determines both the interpretative approach and the sectioning strategy.

### 3.5. Selection of the Biopsy Site

Selecting the biopsy site is one of the most important steps in the procedure. Even when the correct technique is used, an inadequate choice of lesion or location may lead to non-diagnostic or misleading results. The biopsy is the microscopic correlate of the clinical lesion and should be taken from the area most likely to show the most specific histopathological changes. Familiarity with dermatopathology improves lesion selection and has been associated with higher diagnostic accuracy [5].

Several considerations should guide lesion selection and are summarized in Table 1.

#### 3.5.1. Activity and Disease-Driven Site Selection

Active and untreated lesions usually provide the most informative histopathological findings. Late, excoriated, ulcerated, or secondarily infected lesions often demonstrate non-specific reparative changes and should be avoided whenever possible.

The choice of biopsy site depends upon the suspected clinical entity. For instance, in annular or centrifugally expanding dermatoses, such as granuloma annulare or porokeratosis the active border is typically more informative than the central area, which may show only residual or resolving changes. Conversely, in occlusive vasculopathy, an incisional biopsy from the center of a livedoid lesion or a nodule may provide a higher diagnostic yield [17]. In some cases, it is advisable to take more than one biopsy, including diseases in which both lesional and perilesional skin can provide useful information. This is especially true in bullous diseases, where the best site for histology is the lesional site, whereas for DIF, the perilesional biopsy (about 1 cm away from the bulla) is required. For microorganisms detection, the edge of a necrotic skin area provides the best results for culture and special stains. Additionally, in some diseases, biopsy site selection is not guided by clinically visible lesions and must be performed in “normal” appearing skin. In intravascular lymphoma, at least 3 to 4 deep biopsies from the trunk or proximal extremities are recommended. In systemic amyloidosis without clinical lesions, biopsy of subcutaneous fat from the abdomen increases the likelihood of detecting amyloid deposits. Less commonly, in certain metabolic or genetic disorders such as CADASIL, no specific anatomic site is required, and sampling from any accessible area is generally adequate, as diagnostic efficacy depends more on appropriate tissue processing and ancillary techniques than on site selection.

By convention, in small fiber neuropathy the skin biopsy is obtained from the leg, approximately 10 cm above the lateral malleolus, or, in certain cases, 20 cm below the anterior superior iliac spine [18]. Before performing this type of biopsy, it is essential to ensure that the specimen can be immediately frozen or that the fresh sample can be transported rapidly to the laboratory on a saline-moistened gauze. This is critical because nerve tissue is highly fragile, and the diagnosis typically relies on frozen sections; therefore, the tissue can be severely damaged if the procedure is not followed precisely

#### 3.5.2. Timing of Biopsy

As most inflammatory lesions evolve, biopsy timing in inflammatory dermatoses is, usually critical. For example, palpable purpura on the lower extremities is recognized as the most reliable clinical hallmark for most cases of cutaneous leukocytoclastic vasculitis. Histopathological examination of an early lesion (within 24–48 h) of a partially blanchable macule is preferred to detect nuclear dust and vascular damage before reparative changes supervene and for immunofluorescence studies [19]. Conversely, for lupus erythematosus or scarring alopecia, well-established lesions (older than 6 months) may provide the highest diagnostic value for characteristic interface changes and immunofluorescence.

#### 3.5.3. Suspected Depth of Involvement

To determine the required biopsy depth, it is essential to identify the skin layer affected by the dermatosis: the epidermis, dermis, or subcutaneous tissue.

In numerous cutaneous diseases, the superficial dermis contains only secondary or nonspecific findings. Therefore, whenever deep involvement is suspected, the biopsy should include an adequate amount of subcutaneous adipose tissue. This recommendation is particularly important in panniculitis, medium-vessel vasculitis, deep fibrosing disorders, deep lymphoid infiltrates, and certain tumors with predominantly hypodermal growth (Table 2) [20,21]. Panniculitides probably represent the best example of this limitation. Their classification depends largely on the evaluation of the adipose lobules, interlobular septa, and deep vessels [20]. Similarly, scalp biopsies that fail to reach the subcutaneous tissue impede evaluation of the hair bulb (typically located >5 mm deep), precluding the accurate diagnosis of a specific alopecia.

An electric rotary power punch or a double punch technique can be useful alternatives for biopsies of panniculitis, especially in patients with a bleeding diathesis [20,21].

#### 3.5.4. Clinical Heterogeneity

In multifocal or clinically heterogeneous diseases, not all lesions are equal. Selecting the most infiltrated, recently evolving, or clinically typical lesion may increase diagnostic rate. In some scenarios, obtaining more than one biopsy from different sites may be recommended. Adjunctive tools such as dermoscopy and trichoscopy may assist in identifying the most diagnostically relevant area within a lesion. In melanocytic and acral lesions, dermoscopy can help target areas with greater architectural disorder, while in alopecia it may help in locating clinically active sites. Incorporating these tools into biopsy planning may enhance diagnostic precision.

#### 3.5.5. Special Anatomic Sites

Certain anatomic locations require specific consideration. The most relevant sites include the nail unit, scalp, oral mucosa, acral and genital skin, and the lower extremities.

**Figure 1:**
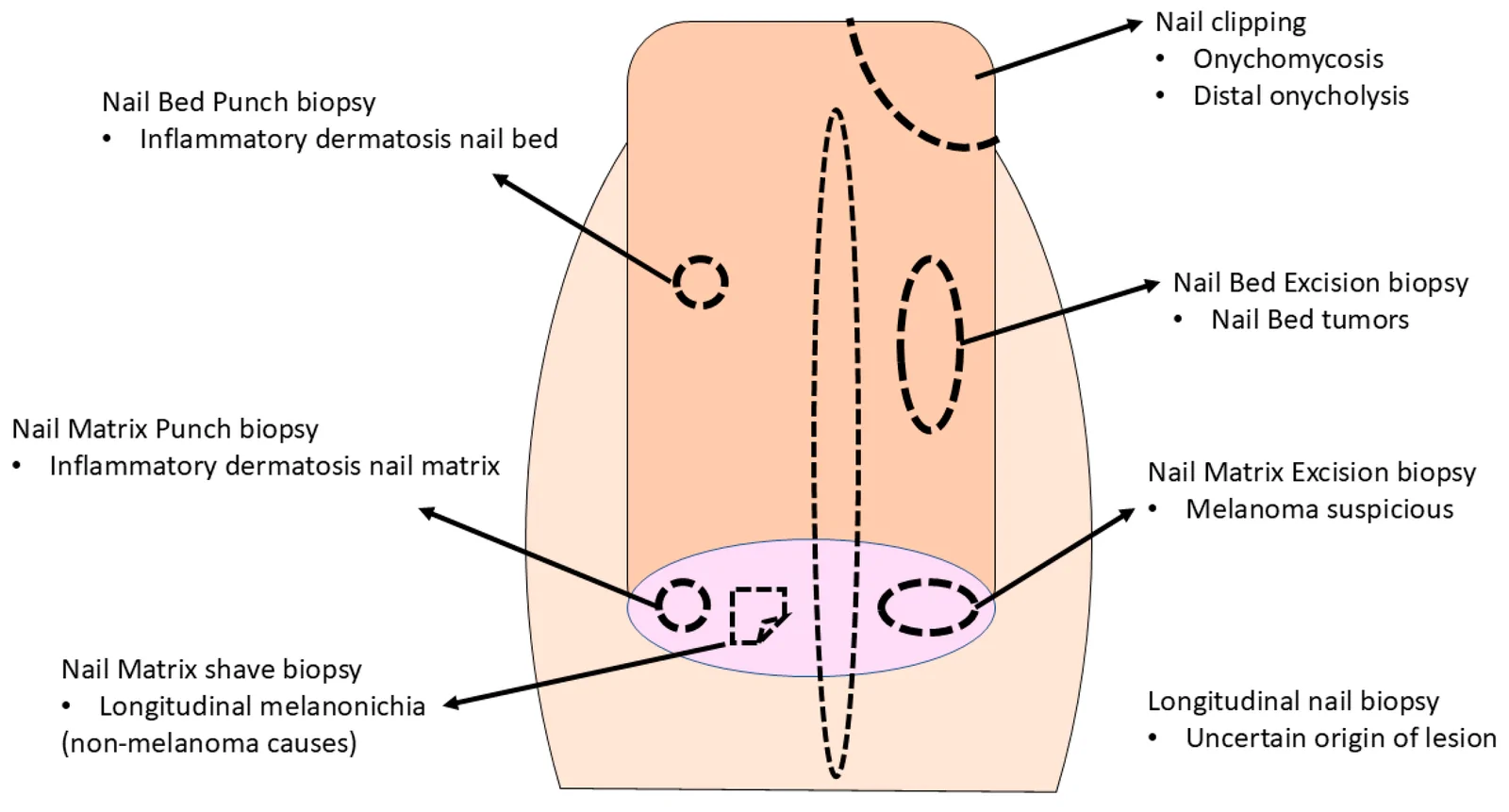
Best procedures for different types of nail biopsies.

Biopsy of the nails deserves special attention due to its complex anatomy and the risk of permanent nail dystrophy. The site and type of nail biopsy (punch, shave, nail clipping or excision) should be selected according to the suspected diagnosis. For instance, pigmented lesions may require matrix biopsy, whereas subungual tumors often necessitate deeper sampling of the nail bed or underlying tissue. Orientation of the specimen may be important, and clear communication with the dermatopathologist is recommended. Furthermore, a precise biopsy technique is critical to minimize permanent nail dystrophy and scarring, ensuring both diagnostic accuracy and optimal functional outcomes for the patient (Figure 1) [22,23].

In alopecia, the biopsy site should correspond to clinically active areas. Sampling from fully scarred or inactive regions may provide only end-stage fibrosis. The HoVert technique (transverse sectioning followed by vertical sectioning of the upper 1 mm section) or the Tyler technique (vertical sectioning of the entire specimen, followed by transverse sectioning of one of the resulting halves) may improve the diagnostic accuracy. Clinical evaluation and, when available, trichoscopy can assist in identifying the optimal site [24,25].

The oral mucosa is prone to artifact and structural distortion; therefore, local anesthetic should not be injected directly into the lesion to avoid tissue distortion. For DIF in suspected autoimmune blistering disorders, biopsies from normal-appearing mucosa 3–4 mm away from the erosion are recommended [20].

Genital and acral skin have site-specific histological features that must be taken into account. Because of difficult wound closure, delayed healing, and increased risk of painful scarring, small incisional biopsies are preferred at these sites. When similar lesions are present elsewhere, less sensitive locations should be selected. On the lower legs, chronic venous insufficiency and edema may produce secondary histologic changes and impair healing. Therefore, biopsy should be performed in areas with minimal trophic changes or at alternative sites whenever possible.

### 3.6. The Importance of the Biopsy Acquisition Technique

The choice of biopsy acquisition technique should be guided by the suspected depth and nature of the pathological process. An inadequate technique compromises diagnostic accuracy, potentially leading to non-specific results or requiring to repeat procedure [6].

#### 3.6.1. Types of Biopsies

Excisional biopsies allow complete removal of the lesion with evaluation of its overall architecture and margins. They are particularly useful when assessment of symmetry, depth, and growth pattern is essential. By providing an intact specimen, excision reduces the risk of sampling error and facilitates accurate histopathological interpretation.

Incisional biopsy, in contrast, is indicated when complete removal is not possible due to lesion size, anatomic constraints, or cosmetic considerations. In these cases, the biopsy should be planned to sample the most representative area. For larger tumors or deep soft tissue processes, the incision, if possible, should be oriented so as not to compromise subsequent definitive surgery.

Both excisional and incisional techniques require careful planning to ensure that adequate depth is achieved and that tissue architecture is preserved. Failure to include the full vertical extent of the lesion may result in underestimation of disease severity or incomplete diagnostic evaluation.

Several methods are available for performing incisional skin biopsies; the most commonly used are punch, curettage, shave and saucerization, and deep incisional (wedge) biopsy [6].

A punch biopsy provides a full-thickness cylindrical specimen of skin and is particularly useful in inflammatory dermatoses in which the diagnostic process involves the dermis or superficial subcutis. A 4 mm punch is generally considered the standard for most inflammatory conditions. Smaller punches (2–3 mm) may be used in cosmetically sensitive areas and pediatric patients; however, they may be insufficient in more complex diseases such as alopecia or panniculitis, where deeper or broader sampling is required. Visualization of subcutaneous fat confirms complete sampling, particularly when deeper inflammatory or neoplastic processes are suspected. Gentle handling of the specimen is crucial to avoid crush artifact and distortion of tissue architecture.

Curettage biopsy is primarily indicated for lesions restricted to the epidermis, such as molluscum contagiosum, seborrheic keratosis, warts, or specific subtypes of carcinoma, such as superficial basal cell carcinoma. Its main advantage lies in its technical simplicity and diagnostic utility; however, its primary drawback is that the specimen is often fragmented, which hinders an accurate evaluation of the surgical margins.

Shave and saucerization biopsies are primarily indicated for superficial and exophytic lesions in which the pathological process is confined to the epidermis or superficial dermis. They are commonly used for seborrheic keratoses, warts, and other benign-appearing epidermal proliferations. This technique is not recommended for lesions suspected of dermal invasion or infiltrative growth, or when accurate assessment of tumor thickness is required, since it may compromise staging and subsequent management.

Deep shave or saucerization biopsy extends into the reticular dermis and may provide a broader and deeper sample while avoiding full excision. When properly performed, it can be adequate for many melanocytic lesions. Nevertheless, insufficient depth or uneven sampling may obscure architectural features and complicate histopathological interpretation.

#### 3.6.2. Number of Biopsies

In most situations, a single biopsy is sufficient to establish the diagnosis. However, in certain clinical scenarios, performing more than one biopsy may improve diagnostic accuracy [6]. For instance, in generalized erythroderma or suspected mycosis fungoides, multiple biopsies from different sites may increase diagnostic sensitivity. In these situations, sampling lesions with different clinical characteristics can improve the probability of establishing a diagnosis.

A similar situation occurs in autoimmune bullous diseases, in which two biopsies are often recommended: one lesional specimen for routine histopathology and a second perilesional specimen for direct immunofluorescence. The lower extremities should be avoided because they are associated with a higher risk of false-negative DIF results.

#### 3.6.3. Importance of Adequate Biopsy Depth

As previously noted, biopsy depth critically influences diagnostic efficacy. A specimen that does not include the anatomical compartment in which the pathological process is located may be insufficient, regardless of the technical quality of histological processing or the experience of the observer [21].

#### 3.6.4. Influence of the Anesthetic Procedure on Biopsy Quality

Skin biopsies are usually performed using local anesthesia, which is generally safe and well tolerated. Lidocaine (1% or 2%) with epinephrine (1:100,000) is the standard. Epinephrine reduces bleeding, keeping the field dry and minimizing hematoma artifact. However, epinephrine should be avoided at sites with compromised end-arterial circulation or when mastocytosis is suspected, as it can cause mast cell degranulation, potentially obscuring the diagnosis.

The technique of anesthetic administration may influence tissue morphology and occasionally introduce histologic artifacts, particularly when the biopsy is obtained directly at the site of anesthetic injection. An excessive volume of anesthetic may produce dermal edema and tissue distortion, which can complicate histopathological interpretation. For this reason, anesthetic infiltration should preferably be performed in the surrounding dermis rather than directly into the lesion whenever possible [26]. In saucerization, a local anesthetic is injected under the lesion using a fine needle; the resulting dermal swelling raises the skin surface, creating an artificial firm papule, allowing the dermatologist to safely control the depth of the cut [27]. However, it can cause morphological distortion if the lesion extends deeper than clinically anticipated, or if excessive anesthetic infiltration alters the superficial dermis.

Both the anesthetic injected solution and the needle itself may alter inflammatory patterns or disrupt delicate structures, particularly in small lesions or inflammatory dermatoses. The anesthetic fluid can separate collagen bundles, creating an appearance that mimics dermal edema. A useful clue for distinguishing this artifact from true edema is the absence of associated lymphatic dilatation in areas affected by anesthetic infiltration. Careful injection technique and the use of minimal anesthetic volume help preserve tissue architecture and improve specimen quality. Finally, topical anesthetics like EMLA (lidocaine and prilocaine mixture) can produce vacuolar changes, acantholysis or artifactual subepidermal blister formation [28,29,30]. In addition, the injection site may show fresh hemorrhage and linear clefts or disruptions within the dermis corresponding to needle tracks. Recognition of these changes is important to avoid their misinterpretation as pathological findings.

### 3.7. Specimen Handling, Fixation and Transport

After the biopsy is obtained, the sample must be managed appropriately to avoid artifacts that may interfere with diagnostic evaluation. Correct fixation, appropriate transport media, and clear communication between the clinician and the pathology laboratory are important steps in this process. Attention should be given when special studies such as microbiological cultures or direct immunofluorescence are required, as these specimens must be handled differently from routine histopathology samples.

#### 3.7.1. Specimen Handling

**Figure 2:**
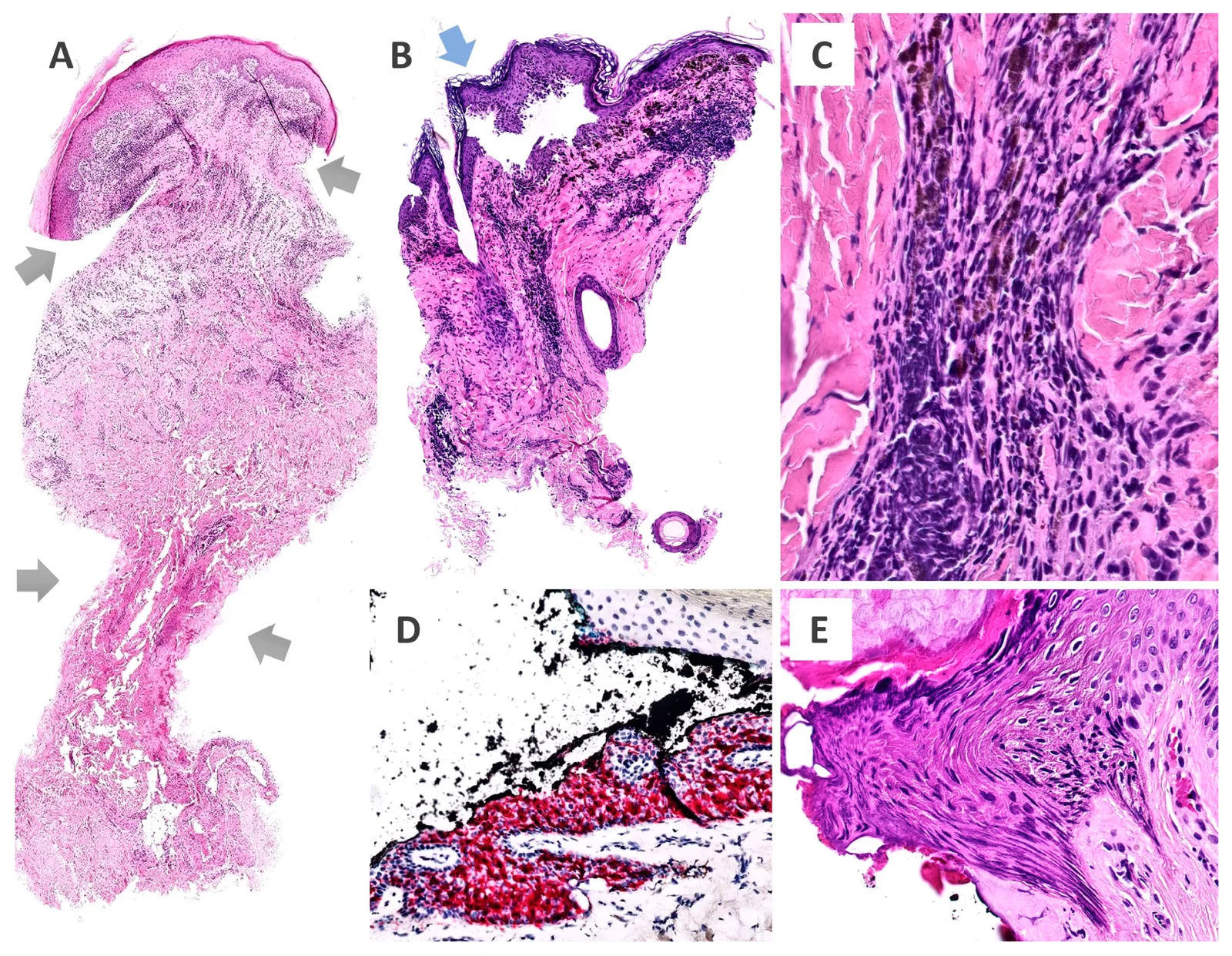
Common artifacts complicating histopathological diagnosis. (**A**) Two foci of crush artifact (gray arrows) caused by excessive pressure applied to a fresh punch biopsy. (**B**) Artifactual dermoepidermal separation (blue arrow) due to improper specimen handling. (**C**) Higher magnification of image (**B**), demonstrating morphologically unrecognizable crushed pigmented and non-pigmented cells. (**D**) Excessive application of marking ink, obscuring structural features and impairing immunostaining visualization. (**E**) Thermal coagulation artifact induced by electrosurgery or laser, producing morphological changes indistinguishable from cytological atypia. This figure is from the Hospital Universitari General de Catalunya laboratory and just illustrates the known pathology.

Immediately after removal, the biopsy specimen should be handled carefully to preserve tissue morphology. If forceps are required to extract the specimen, apply minimal pressure to prevent crush artifacts that can hamper histopathological diagnosis or may mimic fibrotic tumors or scleroderma (Figure 2). The use of a hypodermic needle to extract the punch is an alternative option, although it also carries some risk of tissue damage. The sample should not be placed on dry gauze, as this may lead to desiccation of the tissue and alter histopathological evaluation. If needed, gauze moistened with saline can preserve the tissue’s qualities for a short period, preferably kept at 4 °C.

**Figure 3:**
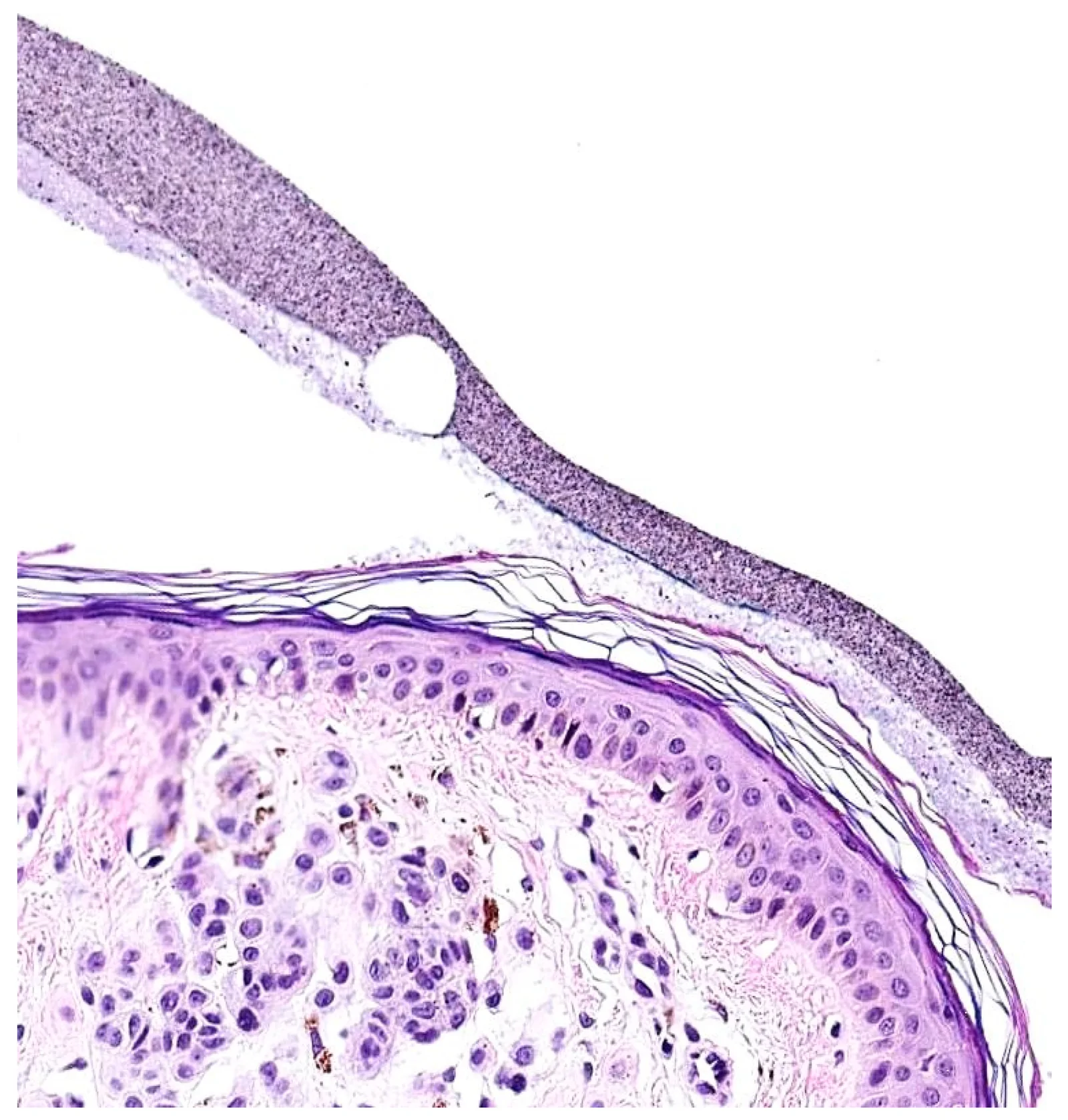
Pigmented lesion oriented using the “derm-dotting” technique with two different nail enamels. Each enamel type displays distinct granularity and can retain a faint hue corresponding to its original color. In this specimen, a paler lacquer located adjacent to the epidermis was used to identify a papular lesion, while a second lacquer with darker granularity marked an area of higher pigmentation. This figure is from the Hospital Universitari General de Catalunya laboratory and just illustrates the known pathology.

When orientation is clinically relevant, the specimen may be marked using sutures, surgical ink, or simple techniques such as derm dotting [1,31]. In derm dotting, areas of interest are marked with nail enamel, which appears under the microscope as an easily recognizable grayish granularity (Figure 3). This technique is simple to perform, improves communication between the clinician and dermatopathologist, and avoids tissue distortion or histological artifacts [1].

When sutures are used for orientation, they should be placed loosely, preferably forming a small loop. Sutures placed too tightly or too close to the specimen edge may damage the tissue and complicate macroscopic processing.

The orientation of exceptionally small specimens may not provide clinically meaningful information. This approach is not a substitute for Mohs micrographic surgery and can unnecessarily interfere with tissue processing. Therefore, specimen marking should be reserved for cases where orientation is technically feasible and expected to directly influence clinical management.

#### 3.7.2. Fixation and Transport to the Histopathology Laboratory

Adequate tissue fixation preserves cellular morphology, prevents autolysis, and maintains tissue integrity for histochemical stains, immunohistochemistry, and molecular testing. Fixation should begin as soon as possible after excision. Preventing tissue desiccation before fixation is particularly important, as drying impairs subsequent penetration of the fixative. If immediate fixation is not possible, the specimen may be wrapped in saline-moistened surgical gauze until it can be placed in fixative. In cases of accidental drying, gentle rehydration with saline before fixation may be beneficial.

Formalin remains the gold-standard fixative for routine histopathology. It preserves tissue architecture and is the preferred fixative for specimens that will undergo paraffin embedding and hematoxylin-eosin staining. Formalin effectively halts autolysis and tissue decomposition while providing sufficient tissue hardening for processing. Nevertheless, it is not ideal for long-term storage and must be handled with caution because of its carcinogenic and potential teratogenic risks [32]. Despite extensive research into alternative fixatives, the authors’ experience is that no currently available substitute offers a superior balance of diagnostic quality, cost-effectiveness, and practicality for routine dermatopathology.

Routine fixation consists of immersion of the specimen in 10% neutral buffered formalin using a container that holds at least ten times the volume of the tissue. A tissue-to-fixative ratio of 1:10 to 1:20 is generally recommended to ensure adequate penetration and tissue preservation [6]. For most specimens, optimal fixation in 10% neutral buffered formalin is achieved within 24 h, with an adequate fixation window of approximately 8–48 h. Smaller specimens, such as punch or shave biopsies, may require shorter fixation periods, whereas larger specimens may tolerate longer fixation. Techniques such as ultrasound-assisted fixation can considerably accelerate the process [33]. Delayed fixation, particularly in warm environments, promotes autolysis and loss of critical histological features, especially nuclear detail. Ideally, specimens should not exceed 1–2 cm in thickness. Thicker samples often require sectioning to facilitate adequate penetration of the fixative.

Overfixation may also adversely affect tissue quality by increasing hardness, complicating microtomy, and producing morphological artifacts. Both under-fixation and overfixation may reduce the sensitivity and specificity of special stains, immunohistochemical studies, and molecular analyses. Several studies have suggested that an initial period of cold formalin fixation at 4 °C followed by fixation at room temperature, may improve preservation of certain biomarkers and enhance subsequent immunohistochemical staining [34]. Following formalin fixation, biopsies may be transferred to 70% ethanol for storage. Although storage in 70% ethanol for up to one month is generally acceptable, prolonged storage may result in alcohol-related fixation artifacts [35].

Tissue deposited into cassettes for paraffin embedding should ideally be no thicker than 3–4 mm to avoid compression artifacts and ensure adequate processing. Fragmented biopsies should be enclosed between cassette sponges or biopsy pads to prevent tissue loss through cassette perforations and to avoid cross-contamination between specimens.

If direct immunofluorescence (DIF) studies are required, the sample should be placed in normal saline at 4 °C when rapid processing by the laboratory is possible. If immediate transport cannot be ensured, the biopsy may be placed in Michel transport medium (MICHTM) [36], which preserves immunoreactants for a longer period and allows delayed processing. If MICHTM is not available, honey has been suggested as an alternative for DIF [37]. In general, saline solution is superior to liquid nitrogen or MICHTM if the sample reaches the laboratory within 48 h.

For frozen sections, as required in Mohs micrographic surgery, skin biopsy specimens should be placed in a specialized water-soluble embedding medium. The most frequently utilized is OCT compound (a mixture of water-soluble glycols and resins) [38].

Alternative fixatives are used for specific applications, such as electron microscopy, including paraformaldehyde, glutaraldehyde, Karnovsky solution, and glyoxal-based solutions. These samples should be stored at 4 °C and sent directly to the histopathology laboratory. If microbiological studies may be required, the specimen should be submitted in a sterile container without formalin, as formalin will destroy microorganisms, following the indications provided by the local microbiology laboratory.

As a general rule, specimens obtained from different anatomical sites should always be submitted in separate, clearly labeled, containers, even when they are considered to correspond to the same pathological process. Normal histological features vary considerably among cutaneous sites and may influence interpretation. For example, in a patient with suspected psoriasis, a biopsy from the palm is more likely to display superimposed eczematous changes than a biopsy from the elbow. Such site-related findings should not necessarily alter the diagnosis. In contrast, the presence of a similar eczematous pattern in an elbow biopsy may warrant reconsideration of the diagnosis. Without knowledge of the biopsy site, these distinctions may be impossible to recognize histologically. In addition, tissue size and shape may become distorted during fixation and processing, making it unreliable to identify specimens sent in the same container based solely on relative dimensions. This is particularly important when the diagnosis may require subsequent procedures, such as wider excision or sentinel lymph node biopsy. Misidentification of specimen origin can lead to unnecessary interventions and patient morbidity.

Finally, thin shave biopsies frequently curl after excision, making proper orientation and evaluation in perpendicular sections difficult. This is especially problematic when assessing melanoma thickness. Whenever possible, shave specimens should be placed flat on a small piece of dry paper or card before immersion in formalin. The fresh tissue adheres immediately to the support, helping maintain proper orientation during processing.

In cases of doubt, clear communication between the clinician and the laboratory is essential to ensure proper specimen handling and avoid loss of diagnostic information.

#### 3.7.3. Common Tissue Artifacts and How to Avoid Them

In addition to the previously mentioned the artifacts secondary to anesthesia, biopsies can present other artifacts that can be divided into mechanical, thermal, chemical and related to the procedure-related artifacts.

Mechanical artifacts can occur in relation with sutures, surgical staples or foreign bodies. Foreign material must be detected during gross examination and removed carefully not to damage the tissue. Informing of their presence in the biopsy request is highly recommended not only to improve the biopsy quality, but also to prevent accidental puncture injury to the prosector. Clinicians should be advised of using very thin thread not tightly knotted or other means for spatial identification to avoid tissue damage. Surgical forceps should be managed with extreme care not to cause crushing or distortion. The compression of the mid-portion of punch biopsies by forceps is especially frequent among non-experienced dermatologists and causes severe diagnostic impairment (Figure 2).

Thermal coagulation artifacts are also very common when lesions are removed with an electric scalpel or laser, potentially impeding the evaluation of surgical margins or the whole lesion in the most severe cases. Epithelial tissues are especially susceptible to damage, showing nuclear enlargement or elongation that can simulate a neoplasia

In tumor re-excisions, ulcerated areas from the previous biopsy site may exhibit black pigmentation due to contamination from silver nitrate sticks. Formalin fixation can also induce similar black artifacts morphologically resembling hemozoin, particularly within hemorrhagic regions. This formalin pigment artifact is exacerbated by prolonged fixation under conditions of high ambient temperature and humidity [39].

Anticoagulant therapy may cause marked dermal hemorrhage that can mimic vasculitis. The disproportionate erythrocyte extravasation, sparse inflammatory infiltrate, and absence of fibrin deposition help distinguish this artifact. However, the anticoagulant use should be mentioned in the biopsy request.

### 3.8. Macroscopic Examination, Orientation and Inking

The first step in the macroscopic examination of the cases is to ensure that the specimen received is correctly identified, corresponds to the patient indicated in the biopsy request form and that its characteristics are consistent with the clinical information. This double check helps ensure the traceability of the cases. At this point, it is necessary to decide which procedure is most appropriate.

Ex-vivo dermoscopy or a stereomicroscopes (dissecting microscope) can help identify lesions, small ulcerations or other features such as the lines of porokeratosis that require a perpendicular sectioning [31]. The derm-dotting technique can also be be used at this stage (Figure 3) [1].

**Figure 4:**
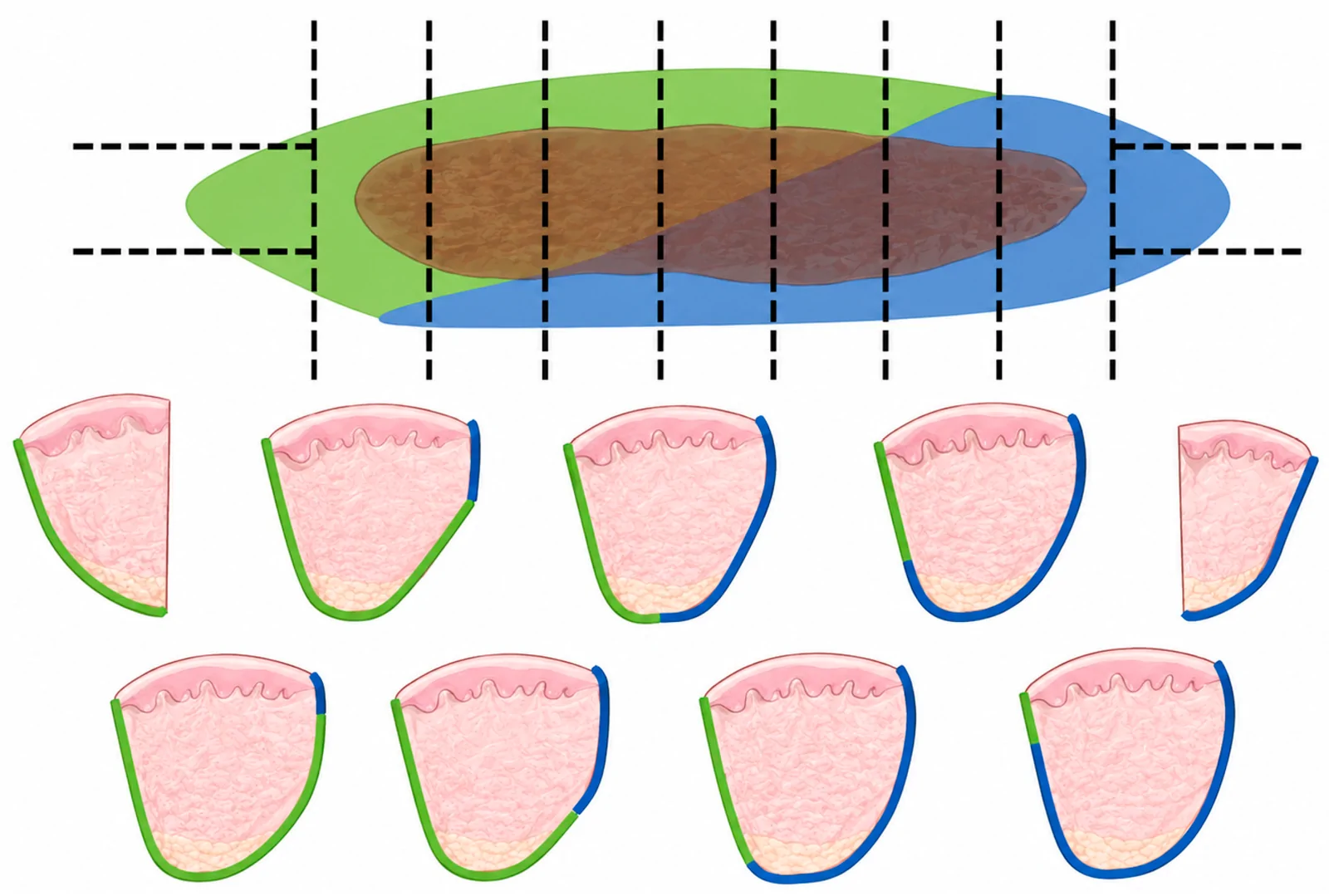
Oblique two-color inking of a skin biopsy. The upper panel illustrates oblique inking of the specimen with two different colors (green and blue), shown here in a pigmented lesion. As illustrated in the lower panels, the relative distribution of the two colors varies progressively in the consecutive sections, allowing their position and orientation within the specimen to be determined. This facilitates the identification and localization of small foci of tumor reaching a margin, allowing their approximate location within the specimen to be reported with reasonable accuracy.

The common presence of fat in cutaneous biopsies can make inking the margins difficult. In addition to drying the tissues before inking, it may be useful to dry the surface and sometimes to briefly soak the surface with a mordant (alcohol, 1% acetic acid or simply vinegar) which will also be used later to stabilize the ink and prevent smearing during subsequent steps. Indelible commercial inks tend to work better than classical Indian ink, although the latter remains satisfactory. Excessive amount of ink can cause it to penetrate into the tissue, making the diagnosis more difficult. Using thin cotton swabs or thin wooden applicators to ink the borders avoids oversoaking and provides greater precision for small specimens. An inexpensive way to identify each section in incisional wedge biopsies without requiring a large number of blocks is to ink the margins obliquely in two colors. The distribution of both colors across the different sections will provides reasonably accurate information about their location in cases of positive margins (Figure 4).

Some biopsies can be easily placed and oriented in the cassette and follow a standard automated embedding procedure to create a paraffin block without human manipulation. However, manually prepared paraffin blocks are necessary for small samples that require precise spatial orientation or sectioning [40].

Specimen orientation may also influence the information obtained. Vertical sections allow optimal assessment of the relationship between the epidermis, dermis, and hypodermis and remain the standard method in most cutaneous diseases. However, particularly in the evaluation of alopecia, transverse or serial sections may provide complementary information by allowing the simultaneous assessment of a greater number of follicular units within a single plane of section [21]. A very recent example is the contribution of horizontal sections to the diagnosis of follicular porokeratosis-associated alopecia [41].

Sectioning of small specimens, such as punch biopsies is easier and more precise at the embedding station after overnight paraffin infiltration. Allowing the tissue to cool slightly at a room temperature provides intermediate hardening. At this point, sections obtained with a sharp blade are cleaner and crushing artifact is avoided. This procedure is highly recommended for the hemisectioning of punch biopsies, and it is especially useful in alopecia, when hemisectioned punch biopsies have to be sectioned again transverselly.

### 3.9. Laboratory Processing Considerations

Skin biopsies are usually small, and the available tissue should be used to maximize the diagnostic yield while preserving sufficient material for ancillary studies if required. However, in most small biopsies, mounting only one to three sections per slide is rarely justified. Especially considering that a 4 mm punch biopsy can provide more than 1200 serial 3-μm sections.

This highlights the paradox of the punch biopsy: the smaller the specimen, the greater the need for multiple initial sections. In very small biopsies, such as 3–4 mm sized punches, diagnostically relevant structures may occupy only a minute fraction of the tissue volume and can therefore be easily missed if only a few superficial sections are examined. Examples include superficial basal cell carcinoma, Fox-Fordyce disease, porokeratosis, lentigo maligna, and syringoma. In these settings, the primary challenge is not the quantity of tissue, but the three-dimensional sampling of the lesion.

**Figure 5:**
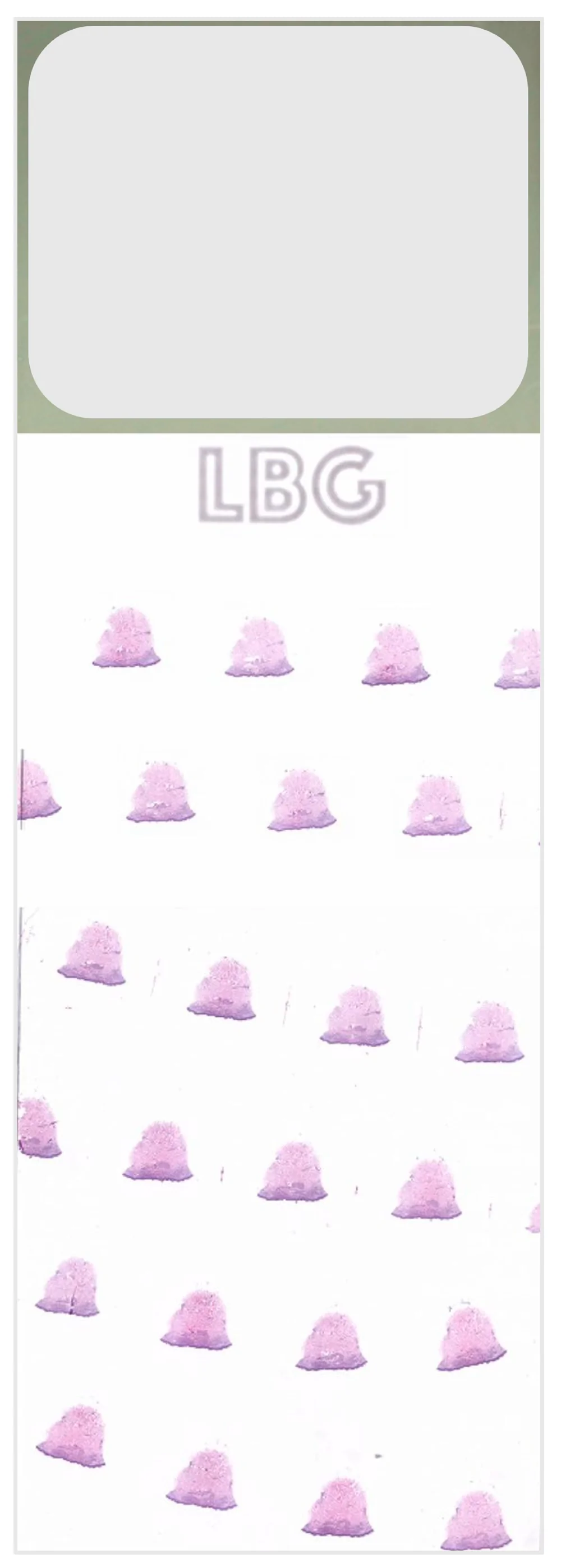
A single slide containing 22 complete 3-μm sections from a punch biopsy represents only 0.066 mm of the sample and can easily be obtained during the initial trimming of the paraffin block on the microtome, without wasting tissue. This approach provides substantially more information than examining only one to three sections and may avoid the need to request additional sections later, which would require further trimming of the block before diagnostically useful sections are obtained, resulting in unnecessary tissue loss. This figure is from the Hospital Universitari General de Catalunya laboratory and just illustrates the known pathology.

Therefore, the diagnostic slide should include as many sections as reasonably possible to reduce non-specific descriptive reports, shorten turnaround time, and improve cost-effectiveness and resource sustainability (Figure 5).

#### 3.9.1. Additional Levels and Deeper Sections

The histological evaluation of a skin biopsy is not always satisfactory based solely on the examination of the initial sections obtained from the paraffin block. Under certain circumstances, obtaining additional levels or deeper sections may improve the overall diagnostic yield of the specimen [42,43,44]. Indeed, one of the maxims shared by many dermatopathologists is that obtaining additional sections is one of the best “special techniques” available for reaching a diagnosis.

Additional levels may also be useful when there is suspicion of orientation artifacts, specimens are superficial, or there are focal lesions that may be incompletely represented in the initial sections. In certain cutaneous neoplasms, deeper sections may reveal diagnostically relevant components that are not evident on the initial sections and may modify the histological classification or the prognostic staging of the lesion, particularly with regard to perineural invasion, which is of critical prognostic importance in cutaneous squamous cell carcinoma [45,46].

An initial slide with as many sections as reasonably possible reduces the need for requesting additional deeper levels later (Figure 5). The routine preparation of multiple slides for all biopsies remains a matter of debate. Although this practice may increase diagnostic accuracy, it also prolongs processing time, increases tissue consumption, and adds to the laboratory workload. For this reason, the decision to request deeper sections should be individualized, based on the initial microscopic findings and the clinical context [42,44]. In this sense, communication between the dermatopathologist and the laboratory plays a key role in the diagnostic process. The implementation of agreed protocols specifying when additional levels should be obtained, how many sections should be examined, and under which circumstances ancillary studies should be requested contributes to improving diagnostic efficiency and optimizing the use of the available tissue [44].

#### 3.9.2. Special Stains and Ancillary Studies

Special stains and ancillary studies should be regarded as tools guided by the diagnostic suspicion rather than as substitutes for an adequate biopsy.

The diagnostic value of histochemical stains is particularly significant for confirming or excluding microorganisms, deposits, collagen alterations, mucin, amyloid, and other specific tissue components (Table 3). However, their use should be judicious, as indiscriminate application increases tissue consumption, processing time, and resource utilization without ensuring a proportional diagnostic benefit [43].

The same principle applies to immunohistochemistry, direct immunofluorescence, microbiological cultures, electron microscopy, and molecular studies (Table 4). Some techniques require specific preservation and handling conditions; therefore, the need for ancillary studies should be considered before the biopsy is performed. For example, DIF is preferably carried out on fresh tissue or using MICHTM [36]. Specimens intended for microbiological culture or electron microscopy likewise require different handling procedures [4,20].

Tissue preservation is becoming increasingly important in contemporary dermatopathology practice. A small biopsy, poor fixation, excessive fragmentation, or unnecessary exhaustion of the specimen through repeated sectioning may limit the possibility of performing additional studies, should new diagnostic questions arise during case evaluation. Therefore, the dermatopathologist and the laboratory must balance the need to obtain immediate diagnostic information with the preservation of sufficient material for potential future ancillary techniques [20,43].

### 3.10. Clinicopathological Correlation

Accurate interpretation of a skin biopsy relies on close clinicopathological correlation. Histopathological findings must always be interpreted in the context of the patient’s clinical presentation, including lesion morphology, distribution, evolution, and prior treatments.

Biopsy interpretation should therefore be understood as an iterative process, in which clinical information guides histological evaluation, and, in turn, histopathological findings refine the clinical differential diagnosis. In some situations, discordance between clinical and histological findings may indicate that the sampled lesion was not representative, or that the disease has evolved since the biopsy was obtained. In such cases, a repeat biopsy may be preferable to overinterpreting limited or inconclusive histological findings. Obtaining a new specimen from a more representative lesion, or from a different stage of disease, may significantly improve diagnostic accuracy.

Clinicopathological correlation is increasingly regarded not only as a communication step but also as a risk-reduction strategy capable of improving diagnostic accuracy and reducing descriptive or discordant reports. It is therefore not confined to the post-analytical phase but is integral to the analytical phase, particularly in challenging melanocytic lesions and within multidisciplinary diagnostic settings. Treating it as merely a validation of a diagnosis at the end of the workflow underestimates its true role. In daily practice, it contributes to the construction of the diagnosis, functioning as an essential component of the analytical interpretative process in which clinical information actively shapes diagnostic reasoning rather than merely confirming the final result.

Effective communication between the clinician and the dermatopathologist is essential in this process. A continuous feedback loop allows refinement of the diagnostic hypothesis and helps determine whether additional studies, deeper sections, special stains, or a repeat biopsy are required. In this regard, clinicopathological conferences represent a valuable tool for discussing challenging cases. These meetings facilitate direct interaction between clinicians and dermatopathologists, allowing integration of clinical and histological data and ultimately improving diagnostic precision and patient management.

### 3.11. Reasons Why Biopsies Are Non-Diagnostic or Suboptimal

A small proportion of skin biopsies do not provide a definitive diagnosis. In such cases, the pathology report is usually limited to a morphological description, a differential diagnosis, or a recommendation to repeat the biopsy. Although this situation may be mistakenly attributed to the limitations of histopathology, it more often reflects a lack of clinical information or problems arising during lesion selection, specimen collection, or tissue processing before microscopic evaluation [20,21,47].

As discussed above, the diagnostic yield of a biopsy depends on the appropriate integration of clinical, technical, and histopathological factors. The selection of a non-representative lesion (Table 1), an inappropriate biopsy technique, the presence of artifacts, or insufficient clinical information may significantly compromise microscopic interpretation and reduce diagnostic value [4,21,48].

It should be emphasized that a non-diagnostic biopsy does not necessarily imply that the biopsy technique was incorrect. Some diseases exhibit nonspecific histological findings, or features that depend on the stage of evolution, making them difficult to recognize even in adequate specimens. Nevertheless, a large proportion of this small group of suboptimal biopsies could be avoided through better biopsy site selection, an adequate biopsy technique, and more comprehensive clinicopathological communication [20,21,47].

Sampling error occurs when the biopsy specimen does not contain the most representative area of the pathological process. Since the dermatopathologist can only evaluate the tissue present in the submitted specimen, the absence of key diagnostic findings cannot be compensated for by additional techniques or by greater diagnostic experience [20,21]. Inappropriate lesion selection is the most common form of sampling error (Table 1) and is not limited to inflammatory dermatoses. In tumor pathology, heterogeneous lesions may contain invasive foci, higher-grade areas, or regression phenomena that are not always represented in limited biopsy specimens, potentially leading to diagnostic underestimation of the lesion [20,47].

The biopsy technique is a fundamental determinant of diagnostic quality [20,21]. Small or superficial biopsy specimens may fail to adequately represent the pathological process. Crush artifacts and thermal coagulation injury alter tissue architecture and may compromise the assessment of surgical margins [48,49]. Similarly, fixation and transport errors may generate artifacts that significantly reduce the diagnostic quality of the specimen (Figure 2) (Table 5) [47,49].

In addition, the lack of clinical information is one of the most underestimated causes of apparently non-diagnostic biopsies. The submission of specimens accompanied only by nonspecific descriptions, such as “skin eruption” or “erythematous plaque,” considerably restricts the dermatopathologist’s interpretative ability. Information such as the patient’s age, anatomical location, duration of the lesion, symptoms, previous treatments, and the clinical differential diagnoses under consideration is essential for placing the microscopic findings into context and narrowing the differential diagnosis [20,21]. The distribution of lesions is of particular importance in dermatology, as numerous diseases share similar histopathological patterns while exhibiting distinctive clinical manifestations. Likewise, clinical photographs may provide essential information regarding morphology, color, and distribution that cannot always be adequately conveyed in written form [21]. Previous treatments may modify the histopathological findings of a lesion and become a cause of inconclusive biopsy results [50]. This is particularly relevant for immunosuppressive agents, which may induce complex lymphoid infiltrates that mimic aggressive lymphomas [21,47]. In psoriasis, for example, therapies targeting IL-23 or IL-12/23 have been shown to modify the histopathological and molecular profiles of lesions [51], whereas anti-TNF agents may induce psoriasiform, lichenoid, or pustular reactions that mimic primary dermatoses [52]. Previous physical procedures may also produce changes that are relevant for histopathological interpretation. Cryotherapy, for example, may induce histological and immunohistochemical alterations in melanocytic lesions that simulate melanoma [53].

In addition, it should be emphasized that the need to repeat a biopsy does not always imply that an initial error has been made; in some cases, it reflects the biological evolution of the disease or the nonspecific nature of its early stages [20,21,50].

### 3.12. Practical Recommendations and Checklist

Most causes of suboptimal skin biopsies are potentially preventable by following a structured “biopsy routine” that addresses the various potential pitfalls discussed throughout this review [4,20,21,48]. This approach should be complemented by direct communication between the clinician and the dermatopathologist [20,21,47].

Both careful planning and the use of checklists or predefined protocols help ensure an appropriate biopsy technique and effective communication between the clinician and the dermatopathologist (Table 6) [20,21,47]. Although these systems were originally developed in other areas of medicine, their underlying principles are fully applicable to dermatological practice [47].

From a practical perspective, measures aimed at optimizing a biopsy can be grouped into three phases: pre-procedural planning, procedural technique, and post-procedural management, including appropriate specimen handling after collection and the provision of adequate clinical information in the request form [21,47,50].

Once the biopsy has been performed, several factors related to tissue identification, handling, fixation, orientation, and transport may significantly influence the quality of the specimen submitted to the laboratory (Table 6).

The pathology request form should be regarded as a clinical communication tool rather than a mere administrative formality. A technically adequate specimen accompanied by insufficient clinical information may significantly reduce diagnostic accuracy and limit clinicopathological correlation (Table 7).

## 4. Future Perspectives

We live in a century filled with new technological opportunities that literally fit in our pockets and can be taken anywhere. This offers both advantages and disadvantages but, above all, requires adequate knowledge and careful management of these resources, which are not always inexpensive.

Clinical photographs are an invaluable tool in dermatopathology, particularly in inflammatory dermatoses. They provide information that is difficult to convey in writing. Their inclusion with the pathology request form improves clinicopathological correlation and may increase diagnostic accuracy [48]. Furthermore, pre-biopsy clinical photography facilitates the correct identification of both the lesion and the biopsy site and should, whenever possible, be incorporated into routine clinical practice in a standardized manner [54]. The increasing availability of digital systems, shared clinical photographs, and telemedicine platforms has enhanced collaboration by providing faster access to relevant clinical information and improving the interpretation of histopathological findings [12,54].

The optimization of skin biopsy is a multidisciplinary process involving clinicians, nursing staff, pathology laboratory technicians, and dermatopathologists. Final diagnostic quality depends on appropriate coordination among all professionals involved in specimen collection, processing, and interpretation. The implementation of standardized protocols, checklists, and effective communication systems helps reduce pre-analytical errors and improve diagnostic results.

**Figure 6:**
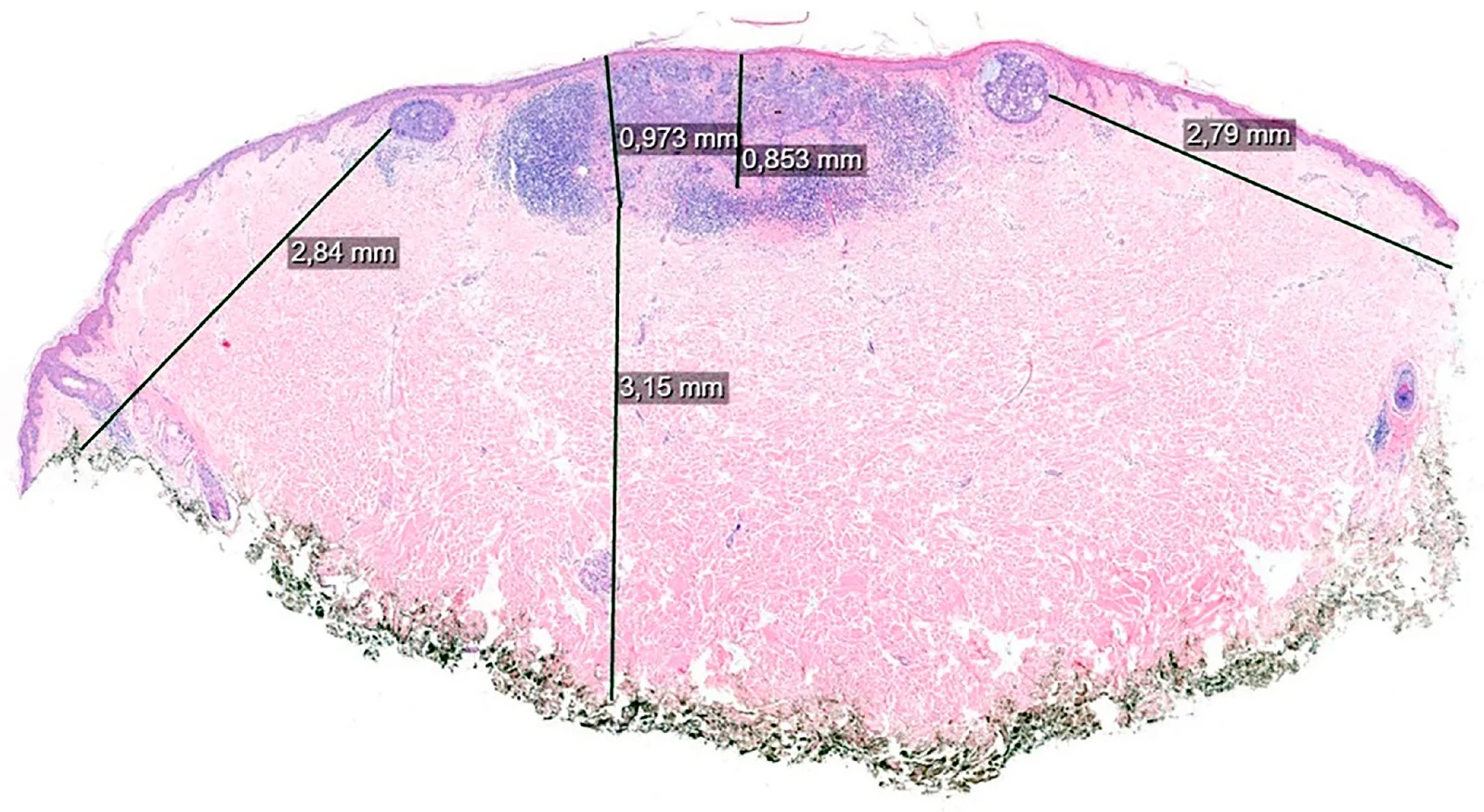
Measurements of interest in the diagnosis of squamous cell carcinoma can be tedious and time-consuming. AI-based algorithms may assist with the automated measurement of tumor dimensions and distances to surgical margins on digitized slides, potentially facilitating standardized and reproducible assessment. This figure is from the Hospital Universitari General de Catalunya laboratory and just illustrates the known pathology.

Skin biopsy remains the cornerstone diagnostic procedure in dermatology. However, digital pathology, teledermatopathology, artificial intelligence, and molecular pathology are progressively transforming the way specimens are obtained, shared, and interpreted. Diagnoses can now be discussed remotely by multiple dermatopathologists, and tumor measurements as well as distances to the surgical margins can be performed with computational assistance on digitized slides (Figure 6). The increasing availability of artificial intelligence tools for selected diagnostic tasks represents an important development that may become increasingly integrated into routine clinical practice. However, the performance of such systems depends on the specific task, training data, validation setting, and quality of the underlying images, and they should currently be regarded as complementary to expert interpretation rather than as replacements for it [55,56].

### 4.1. Digital Pathology and Image Sharing

Digital pathology represents one of the most important transformations in contemporary dermatopathology. Whole-slide imaging (WSI) now plays an increasing role in routine primary diagnosis, telepathology, and reorganization of centralized laboratory workflows. In addition, facilitates remote consultations and expert second opinions without the logistical delays or risks associated with shipping glass slides [56,57]

From a biopsy workflow perspective, digital pathology can bring together information generated at different stages of the diagnostic process. Digitized slides can be reviewed alongside clinical photographs, dermoscopic images, previous biopsies, and ancillary studies on a single interactive platform, strengthening clinicopathological correlation and facilitating assessment of specimen adequacy and communication when additional sampling or deeper sections are being considered. It also supports teaching activities, quality assurance programs, and long-term digital archiving, contributing to improved diagnostic reproducibility [55] and facilitating retrospective audits.

Modern digital workflows also serve as the foundation for integrating computational pathology and artificial intelligence (AI). Algorithms designed to assist in pattern recognition, such as automated cell counting, immunostaining quantification, margin assessment, and pre-diagnostic triage for common lesions are improving diagnostic efficiency and reducing observational variability.

It is foreseeable that, over the coming years, digital pathology will transition from a complementary tool to the primary environment for dermatopathology practice. While optical microscopes may remain valuable for specific ancillary checks, digital workstations are rapidly becoming the primary tool for specimen evaluation.

### 4.2. Artificial Intelligence and Diagnostic Assistance

Artificial intelligence is emerging as an increasingly important support tool in dermatopathology. Systems currently under development are capable of identifying histopathological patterns, quantifying biomarkers, and assisting with specific diagnostic tasks, thereby potentially improving reproducibility and reducing interobserver variability [58].

With regard to biopsy quality, these technologies may help identify inadequate specimens, detect technical artifacts, and highlight areas of diagnostic interest, and will most likely draw attention to these limitations during the final interpretation.

Although artificial intelligence is currently regarded as a complement to the expertise of the dermatopathologist rather than a replacement, its role in diagnostic practice is likely to increase. Studies evaluating AI in histopathological image analysis have demonstrated promising performance in selected diagnostic tasks, including the recognition and quantification of morphological features [59,60,61,62,63]. However, performance varies according to the disease, dataset, task, and validation setting. Currently, these systems should be considered complementary tools rather than substitutes for expert histopathological diagnosis.

Beyond the potential benefits of AI, important issues remain to be addressed, including costs and equitable access, ethics and liability, the need for appropriate external quality assessment, and the risk that excessive reliance on automated systems could weaken human reasoning, curiosity, and diagnostic independence.

### 4.3. Teledermatopathology and Pre-Biopsy Consultation

Teledermatopathology represents a natural extension of digital pathology, enabling the remote review of skin biopsies, the acquisition of second opinions, and consultations among geographically distant specialists [56,57]. It can improve access to specialized expertise in resource-limited settings and help reduce diagnostic disparities among different healthcare institutions [56].

### 4.4. Integration of Molecular Pathology into Routine Practice

The increasing incorporation of molecular techniques is expanding the amount of information that can be obtained from a skin biopsy. Genetic, molecular, and clonality studies are playing an increasingly important role in the diagnosis and classification of numerous cutaneous diseases, including melanocytic tumors, cutaneous lymphomas, histiocytoses, and a variety of genodermatoses [64,65,66,67,68,69,70].

This has led to the reclassification of numerous entities, not only neoplastic but also inflammatory, based primarily on molecular rather than morphological criteria. Examples include the newly recognized histiocytosis defined by histiocytic gene fusions, the molecular pathways underlying the different melanoma subtypes, and newly recognized inflammatory disorders defined by the identification of pathogenic mutations [65,68,71,72,73,74,75].

Far from representing mere genetic curiosities, many of these “molecular” diagnoses have important therapeutic implications, making it increasingly necessary for dermatopathologists to incorporate these diagnostic tools into routine practice, despite their not always being readily available [64,70,72,76].

In the field of inflammatory pathology, spatial transcriptomics (the topographical identification of transcribed RNA within a biopsy section) is redefining our understanding of the pathogenesis of numerous diseases, broadening our knowledge of these conditions and potentially contributing to improved disease classification and therapeutic management [77,78,79,80,81].

### 4.5. Standardization of Biopsy Protocols

As discussed throughout this review, the diagnostic quality of a biopsy is influenced by multiple factors, and considerable variability exists among institutions with respect to each of these aspects. Consequently, one of the main priorities for the future is the development of standardized protocols establishing minimum quality criteria for the acquisition, processing, and documentation of skin biopsy specimens [82,83].

These protocols may be either general or tailored to specific situations, such as inflammatory diseases, alopecia, vasculitis, panniculitis, melanocytic tumors, cutaneous lymphomas, or immunofluorescence and molecular pathology studies [84,85,86,87,88]. Their purpose would not be to limit clinical flexibility, but rather to reduce avoidable variability and promote a more homogeneous and reproducible practice [82,83].

### 4.6. Towards an Integrated Model of Dermatopathology

Dermatopathology is evolving towards an increasingly integrated model in which clinical information, dermoscopy, digital pathology, immunohistochemistry, molecular biology, and artificial intelligence can contribute jointly to the diagnostic process.

Nevertheless, (at least for the time being), it is reassuring to know that hematoxylin and eosin staining remains the central foundation upon which all these tools are built. Indeed, the more sophisticated diagnostic techniques become, the more important the quality of the initial biopsy specimen becomes as the cornerstone of the final diagnosis.

## 5. Conclusions

Skin biopsy is one of the most important diagnostic procedures in dermatology and continues to play a leading role in the dermatopathology practice. Despite the remarkable advances achieved in immunohistochemistry, molecular pathology, genomic sequencing, digital pathology, and artificial intelligence, the quality of the biopsy specimen remains a main determinant of diagnostic accuracy. No technology, however sophisticated, can fully compensate for the limitations of a poorly selected, inadequate, or improperly handled biopsy specimen.

Throughout this review, we have sought to demonstrate that biopsy quality depends on numerous factors acting in a sequential and interdependent manner. Weakness at any point in this chain may significantly compromise the final outcome. Consequently, skin biopsy should not be regarded as an isolated procedure but rather as a multidisciplinary process, with final diagnostic quality depending on the coordinated contribution of multiple professionals.

We wish to emphasize that many of the diagnostic limitations encountered in routine practice do not arise from complex or unavoidable problems, but rather from seemingly minor errors occurring during specimen collection or handling. Considering that correcting these small deficiencies may reduce the need for repeat biopsies, unnecessary ancillary studies, and diagnostic delays, it is well worth paying closer attention to these fundamental aspects of the biopsy procedure.

Although the progressive incorporation of new technologies offers extraordinary diagnostic opportunities, all of them ultimately depend on the quality of the initial biopsy specimen.

Among all the factors discussed, none is probably of greater practical importance than communication between the clinician and the dermatopathologist. Clinicopathological correlation remains one of the fundamental principles of dermatopathology and is still, in many cases, the key to achieving an accurate diagnosis. Modern technologies are particularly valuable for strengthening this communication through rapid access to clinical information and high-quality clinical images.

Looking ahead, we should promote the implementation of more standardized protocols, digital clinical decision-support tools, and increasingly closer models of collaboration among specialties. Ultimately, a skin biopsy represents far more than a fragment of tissue placed in a container of formalin. Behind every specimen there is a patient experiencing suffering, uncertainty, concern, or anxiety. The best dermatopathology begins long before the specimen reaches the microscope. It begins at the moment the clinician decides which lesion to biopsy, how to perform the procedure, and what clinical information will accompany the specimen. Maintaining this perspective is essential to ensure that every biopsy achieves its maximum diagnostic potential.

## Figures and Tables

**Table 1:** Optimal biopsy sites for the most critical to suspected diagnoses, listed in alphabetical order.

Suspected Diagnosis	Where/How to Biopsy	Notes/Caveat
Alopecia (non-scarring)	Active area of hair loss	Avoid end-stage longstanding alopecia
Alopecia (scarring)	Active lesion of recent onset with terminal hairs	Avoid end-stage alopecia
Alport syndrome	Normal skin	Evaluate type IV collagen abnormalities
Atypical hemolytic uremic syndrome	Normal skin	May show thrombotic microangiopathy
Autoimmune bullous diseases and vasculitis: *DIF	Perilesional normal-appearing skin (<1 cm from blister)	Avoid necrotic or eroded skin. Send in saline or *MICHTM (never formalin)
Autoimmune bullous diseases: routine histology	Edge of an early vesicle or bulla, including adjacent intact skin	Avoid ulcerated or late lesions Never send the blister roof only
CADASIL	Random normal skin	Electron microscopy or immunostaining for NOTCH3
Calciphylaxis	Deep biopsy	Be sure to include enough fat tissue
Connective tissue diseases	Established active lesion (>6 months)	Avoid old lesions. Consider DIF for lupus erythematosus
Cutaneous lymphoma	Untreated active lesions. For Intravascular large B-cell lymphoma random normal skin (≥2–3 biopsies)	Deep incisional biopsy preferred over superficial punch. Avoid necrotic excoriated skin
Dermatofibrosarcoma protuberans	Deep incisional biopsy including subcutis	Include subcutaneous fat tissue
Epidermolysis bullosa	Fresh blister	Select an early lesion (<24 h)
Epidermolysis bullosa (*DIF mapping)	Fresh or induced blister	Avoid broken blisters
Keratinocytic intraepidermal neoplasia (Bowen disease and actinic keratosis)	Incisional biopsies should target the thickest or most clinically atypical area	Include epidermis and superficial dermis; Hyperkeratosis (corneal layer) may exceed 1 mm.
Lentigo maligna	Multiple areas or broad shave including different colors	Several specimens from different areas increase sensitivity
Lysosomal storage disorders	Normal skin (often axillary)	Ultrastructural abnormalities in adnexal cells
Melanocytic lesions (suspected melanoma)	Complete excisional biopsy when possible	For incisional specimens, select region most suggestive of deep invasion (e.g., nodular components or ulceration)
Molecular testing	Representative viable lesional tissue	Avoid necrotic areas. Histological sections are necessary to confirm the tumor presence
Morphea	Inflammatory border including adjacent indurated skin	If possible, include both the lilac ring and the sclerotic area in the same specimen
Mucosal lesions	Scalpel incisional or punch-biopsy	Avoid artifacts from electrosurgery and intralesional anesthetic injection
Panniculitis	Deepest active nodule including subcutis	Deep incisional biopsy including subcutis. If punch is used, must be ≥6 mm and consider punch on punch
Pigmentary disorders (vitiligo, hypopigmentation)	Sample normal and affected skin from the edge of lesion or take two samples	Compare the differences, if possible within same specimen
Porphyria cutanea tarda	Intact blister on sun-exposed skin	Diagnostic features can be lost in old blisters
Pseudoxanthoma elasticum	Flexural normal skin	Von Kossa stain confirms calcium deposition
Small fiber neuropathy	Distal leg normal skin	Search for reduced intraepidermal nerve fiber density
Stevens-Johnson syndrome/*TEN	Early lesions including full epidermal thickness	Late lesions may mimic other entities
Suspected bacterial, mycobacterial or fungal infection	Active untreated lesion	Consider sending fresh sterile tissue for microbiological study
Systemic amyloidosis	Abdominal fat pad or affected skin (purpura, plaques, or waxy lesions)	Higher yield than random normal skin
Vasculitis	Early purpuric lesion (<24–48 h for DIF; established lesion for H&E)	Timing and lesion age are critical. Avoid late lesions. Consider *DIF

*DIF: direct immunofluorescence; *TEN: toxic epidermal necrolysis; *MICHTM: Michel transport medium.

**Table 2:** Optimal biopsy depth according to the main diagnostic compartment.

The Biopsy Should Preferably Include…	Representative Diseases/Clinicopathological Settings
Epidermis and superficial dermis	Psoriasis, lichen planus, pityriasis lichenoides, superficial dermatophyte infection, viral exanthems, interface dermatitis with mainly superficial involvement
Full-thickness dermis	Granuloma annulare, cutaneous sarcoidosis, necrobiosis lipoidica, interstitial granulomatous dermatitis, urticaria/urticarial vasculitis, drug eruptions
Deep dermis	Morphea, scleromyxedema, deep granulomatous dermatitis, perforating disorders, adnexal tumors with deep extension
Deep dermis and superficial subcutis	Medium-vessel vasculitis, cutaneous polyarteritis nodosa, livedoid vasculopathy, deep fungal/mycobacterial infection, lupus panniculitis, morphea profunda
Generous subcutaneous tissue	Panniculitis, erythema nodosum, subcutaneous panniculitis-like T-cell lymphoma, pancreatic panniculitis, alpha-1 antitrypsin deficiency panniculitis, calciphylaxis
Hair follicle-bearing skin extending into subcutis	Alopecia, folliculitis decalvans, dissecting cellulitis, hidradenitis suppurativa, follicular tumors, folliculotropic mycosis fungoides
Lesional edge including adjacent normal skin	Blistering diseases for routine histology, interface dermatitis, porokeratosis, annular lesions, vasculitis, ulcers
Perilesional normal-appearing skin	Direct immunofluorescence for autoimmune blistering disease, lupus erythematosus, vasculitis, dermatitis herpetiformis
Favor excisional or deep saucerization	Melanocytic tumors, suspected melanoma, adnexal neoplasms, cutaneous lymphoid infiltrates, Merkel cell carcinoma, poorly differentiated tumors
Edge and base of the ulcer	Pyoderma gangrenosum, infectious ulcers, vasculitic ulcers, neoplastic ulcers, hypertensive ischemic ulcer (Martorell ulcer)

**Table 3:** Main histochemical stains in dermatopathology and their diagnostic utility.

Histochemical Stain	Main Diagnostic Utility in Dermatopathology
PAS	Fungi, basement membrane thickening, glycogen, and some adnexal tumors.
PAS-diastase	Distinguishes glycogen from diastase-resistant material; useful in storage/metabolic disorders and selected tumors.
Grocott (GMS)	Fungi, Pneumocystis, and some filamentous bacteria such as Nocardia.
Ziehl–Neelsen	Acid-fast mycobacteria, particularly when bacillary load is high.
Fite-Faraco	Leprosy and other partially acid-fast organisms.
Gram	Bacterial infections including impetigo, ecthyma, botryomycosis, and suppurative lesions.
Warthin–Starry/Steiner	Spirochetes and selected small bacteria (e.g., syphilis, bacillary angiomatosis).
Alcian blue	Dermal mucin in lupus erythematosus, dermatomyositis, scleromyxedema, and myxoid tumors.
Colloidal iron	Demonstration of dermal mucin, especially in connective tissue diseases.
Movat pentachrome	Elastic fibers, mucin, collagen, and fibrin; useful in vascular and connective tissue disorders.
Verhoeff–Van Gieson/Orcein	Elastic fibers in pseudoxanthoma elasticum, anetoderma, solar elastosis, and vascular lesions.
Masson trichrome	Collagen deposition and fibrosis in morphea, scars, and sclerosing disorders.
Congo red	Amyloid deposits in localized or systemic amyloidosis.
Von Kossa	Calcium deposition in calcinosis cutis and calciphylaxis.
Alizarin red	Calcium deposits; complementary to Von Kossa.
Perls (Prussian blue)	Hemosiderin in pigmented purpuric dermatoses, stasis dermatitis, and hemorrhagic lesions.
Fontana–Masson	Melanin in pigmentary disorders and melanocytic lesions.
Oil Red O/Sudan stains	Neutral lipids (requires frozen tissue); useful in xanthomas and lipid-rich lesions.
Toluidine blue/Giemsa	Mast cells, selected parasites, and inflammatory patterns.
Luxol fast blue	Myelin and nerve assessment in selected neural disorders and leprosy.

**Table 4:** Ancillary diagnostic techniques in dermatopathology: indications and specimen handling.

Technique	When Should It Be Considered?	Specimen Handling
Immunohistochemistry (IHC)	Classification of inflammatory dermatoses; melanocytic lesions; epithelial, adnexal and soft tissue tumors; cutaneous lymphomas; selected infectious agents; prognostic and predictive biomarkers.	Formalin-fixed paraffin-embedded (FFPE) tissue. Standard ancillary technique in routine dermatopathology.
Direct immunofluorescence (DIF)	Autoimmune blistering diseases; lupus erythematosus; dermatitis herpetiformis; cutaneous vasculitis; selected immune-mediated disorders.	Perilesional, non-ulcerated skin. Fresh tissue in Michel’s transport medium (or equivalent). Never place in formalin.
Microbiological cultures	Suspected bacterial, mycobacterial, fungal and selected viral infections.	Fresh sterile tissue. Do not place in formalin. Send promptly to the microbiology laboratory.
Electron microscopy (EM)	Selected genodermatoses, blistering diseases, ciliary disorders, storage diseases and occasional research applications.	Immediate fixation in glutaraldehyde. Reserved for selected indications.
Molecular pathology	Gene rearrangements, mutations, copy number alterations, clonality studies, pathogen detection and gene-expression profiling.	FFPE tissue is suitable for most assays; fresh tissue may be preferable for selected techniques. Ensure adequate lesional cell content.
Spatial transcriptomics/multiplex tissue imaging	Selected research applications; complex inflammatory dermatoses; tumor microenvironment studies; biomarker discovery. Not recommended for routine diagnosis.	Fresh frozen tissue is generally preferred; FFPE-compatible platforms are increasingly available. Tissue handling must follow platform-specific requirements to preserve RNA quality.

**Table 5:** Ten common biopsy mistakes that reduce diagnostic yield.

Common Mistake	Potential Consequence
Superficial biopsy for suspected panniculitis	Subcutaneous tissue absent; diagnosis may be impossible.
Submitting a DIF specimen in formalin	Direct immunofluorescence cannot be performed.
Biopsying an old vasculitic lesion	Characteristic vascular changes may have disappeared.
Sampling only the ulcer base	Predominantly non-specific necrosis and inflammation.
Obtaining cultures after prolonged antibiotic therapy	False-negative microbiological results.
Biopsying only the blister roof	Loss of the diagnostic dermoepidermal interface.
Providing insufficient clinical information	Limited clinicopathological correlation and lower diagnostic accuracy.
Excessive tissue crush with forceps	Cytological distortion and architectural artifacts.
Electrocautery applied to diagnostic tissue	Thermal artifact compromising histological interpretation and margin assessment.
Tiny or fragmented biopsy specimen	Poor orientation and inadequate assessment of lesion architecture.

**Table 6:** Biopsy optimization checklist.

Phase	Recommendations
Specimen identification	• Confirm the patient’s identity. • Verify consistency between specimen container and pathology request form. • Label the container immediately after specimen collection. • Avoid unlabeled containers or those labeled retrospectively.
Tissue handling	• Minimize direct manipulation of the specimen. • Avoid crush artifact caused by traumatic forceps. • Use appropriate instruments to reduce mechanical artifacts. • Preserve specimen integrity and avoid unnecessary fragmentation.
Fixation	• Place the specimen in fixative immediately after collection. • Use an adequate volume of fixative (ideally ≥10 times the tissue volume). • Ensure the specimen is completely submerged. • Avoid prolonged delays before fixation. • Use appropriate transport media when special techniques are required (e.g., DIF).
Orientation	• Mark specimen orientation when diagnostically relevant. • Use sutures, inks, or conventional markers to identify margins. • Clearly inform the laboratory of the meaning of each marker.
Transport	• Ensure rapid and safe transport to the laboratory. • Avoid extreme temperatures. • Prevent specimen loss and fixative leakage. • Verify correspondence between each container and its pathology request form.
Final verification	• Confirm that the specimen is correctly identified, adequately fixed, and accompanied by the necessary clinical documentation before submission to the laboratory.

**Table 7:** Recommended clinical information in the pathology request form.

Information	Diagnostic Value
Age and sex	Provide clinical context for histopathological findings and help refine the differential diagnosis.
Relevant medical history	Facilitate interpretation of lesions associated with systemic diseases, immunosuppression, or previous malignancies.
Precise anatomical location	Many diseases show characteristic anatomical distributions with specific diagnostic implications.
Duration of the lesion	Histopathological findings may vary considerably according to the stage of lesion evolution.
Growth rate and recent changes	Help interpret inflammatory, infectious, and neoplastic processes.
Associated symptoms	Pruritus, pain, or other symptoms may help guide the differential diagnosis.
Clinical description	Should include morphology, color, size, number of lesions, and distribution.
Clinical differential diagnosis	Allows integration of histopathological findings into the clinical context and optimizes clinicopathological correlation.
Previous treatments	Corticosteroids, immunosuppressants, biologics, antibiotics, cryotherapy, laser therapy, and other procedures may modify histopathological features.
Dermoscopic findings	Particularly useful in melanocytic lesions and cutaneous tumors.
Relevant ancillary studies	Laboratory tests, microbiology, immunology, or imaging studies may provide decisive information for interpretation.

## Data Availability

No new data were created or analyzed in this study. Data sharing is not applicable to this article.

## Abbreviations

The following abbreviations are used in this manuscript:

DIF	Direct immunofluorescence
MICHTM	Michel transport medium
TEN	Toxic epidermal necrolysis
WSI	Whole-slide imaging
